# Interfacial Microstructure Evolution and High-Speed Ball Shear Fracture of SAC305/Cu-20wt%Zn Solder Joints Under Isothermal Aging

**DOI:** 10.3390/ma19143138

**Published:** 2026-07-22

**Authors:** Jae-Yong Park, Sehoon Yoo

**Affiliations:** 1 Division of Materials Science & Engineering, Hanyang University, Seoul 133-791, Republic of Korea; pjy0913@hanyang.ac.kr; 2 Advanced Packaging Integration Center (APIC), Korea Institute of Industrial Technology, Incheon 406-840, Republic of Korea

**Keywords:** Sn-Ag-Cu solder, Cu-Zn layer, electroless Ni immersion gold, intermetallic compound, shear strength, high-speed shear test

## Abstract

The interfacial microstructure evolution and high-speed ball shear reliability of SAC305 (Sn-3.0Ag-0.5Cu)/Cu-20wt%Zn solder joints were systematically investigated after isothermal aging at 180 °C for up to 250 h. SAC305/electroless nickel immersion gold (ENIG) joints were used as a comparative reference. Unlike prior studies that benchmarked Cu-Zn against bare Cu, this work directly compares the two systems, establishing ENIG as the industrially relevant reference. Cu_6_(Sn,Zn)_5_ was identified as the dominant intermetallic compound (IMC) phase at the SAC305/Cu-Zn interface by SEM/EDS analysis. At the SAC305/ENIG interface, (Cu,Ni)_6_Sn_5_ formed as the dominant IMC phase, accompanied by a P-rich layer at the (Cu,Ni)_6_Sn_5_/Ni(P) boundary. The IMC thickness of SAC305/Cu-Zn joints increased from approximately 2.50 μm in the as-reflowed condition to 3.41 μm at 250 h, consistently exceeding that of SAC305/ENIG joints (1.94–2.25 μm) throughout aging. Despite this, the high-speed ball shear strength of SAC305/Cu-Zn joints was equivalent or superior to that of SAC305/ENIG joints at all aging durations. Fractographic analysis confirmed that the P-rich layer in ENIG joints acted as a preferential crack propagation path under impact loading, driving the brittle fracture ratio to approximately 75% at 250 h—compared to approximately 47% in SAC305/Cu-Zn joints. These results demonstrate that Cu-Zn electroplated from a neutral pyrophosphate-based bath constitutes a highly reliable wetting layer, offering impact reliability equivalent or superior to that of the conventional ENIG surface finish.

## 1. Introduction

The rapid expansion of AI-driven data centers has increased both power density and operating temperature in server and networking packages. In high-performance computing (HPC) environments, package-level solder ball interconnections—typically Sn-Ag-Cu (SAC) alloys at ~300 μm pitch—are continuously exposed to elevated temperatures and repeated thermal cycling. Under these conditions, intermetallic compound (IMC) growth at the solder/pad interface is a well-established cause of mechanical failure under impact and fatigue loading. Optimizing surface finish design to improve interfacial reliability has therefore become a critical challenge in next-generation data center packaging.

The long-term reliability of package-to-board solder joints in FCBGA and 2.5D/3D integrated packages is governed by the IMC layer formed at the solder ball/surface finish interface during reflow. The composition, thickness, and morphology of this layer evolve during thermal aging and directly determine joint mechanical integrity.

Among available surface finishes, electroless nickel/immersion gold (ENIG) has been most widely adopted in electronics packaging. Its advantages include excellent solderability, flat surface topography, and long shelf life [1]. The electroless Ni(P) layer acts as a diffusion barrier between the SAC solder and the underlying Cu pad, suppressing rapid Cu–Sn IMC growth during reflow and aging. However, ENIG has well-known reliability limitations. During electroless Ni deposition, a phosphorus (P)-enriched layer inevitably forms at the Ni(P)/IMC interface. Beneath this P-rich layer, a nano-void-containing Ni-Sn-P transition layer develops at the (Cu,Ni)_6_Sn_5_/P-rich layer boundary. This interfacial region is inherently susceptible to brittle fracture and provides a preferential crack propagation path under impact loading [2,3,4]. The well-documented “black pad” defect—caused by hyper-corrosion of the Ni(P) surface during immersion Au deposition—can also severely degrade solderability and joint strength without any visible indication prior to assembly [5,6]. Furthermore, the ferromagnetic nature of the Ni(P) layer introduces magnetic loss and reduces skin depth at high frequencies. This poses increasing signal integrity challenges as AI server interconnects operate in the multi-gigahertz regime [7]. To eliminate these Ni-related limitations, nickel-less surface finishes such as direct electroless gold (DEG) and electroless palladium immersion gold (EPIG) have recently been investigated, and have shown long shelf life comparable to or better than Ni-based finishes despite thicker Cu–Sn IMCs [4,8]. The electroplated Cu-Zn wetting layer studied here belongs to the same nickel-less category.

Building on early studies of Zn-modified Cu substrates, Kim et al. [9,10] demonstrated that an electroplated Cu-Zn alloy wetting layer markedly improves drop impact reliability of SAC solder joints. Zn addition suppressed large Ag_3_Sn plate formation in the solder bulk—a preferential crack path under dynamic loading—and retarded Cu_6_Sn_5_ IMC growth at the solder/Cu-Zn interface. Unlike the SAC/Cu system, neither Cu_3_Sn nor Kirkendall microvoids were observed during solid-state aging. This eliminated a major source of interfacial weakening [9,10].

The interfacial reaction mechanism in the SAC/Cu-Zn system is now well established. During reflow, Zn dissolved from the Cu-Zn layer accumulates preferentially at the interfacial IMC. Rather than occupying the Cu sublattice, Zn partially substitutes for Sn on the Sn sublattice of η-Cu_6_Sn_5_, forming Cu_6_(Sn,Zn)_5_. FE-EPMA compositional data confirm this: as Zn content in the IMC increases, Sn content decreases correspondingly, while Cu sublattice occupancy remains constant at ~54 at.% [11]. Because Zn reduces the thermodynamic driving force for Cu_3_Sn nucleation, Cu_3_Sn is completely suppressed at the SAC/Cu-Zn interface [11,12]. Microvoid and Kirkendall void formation are also absent at all aging durations. This is attributed to Zn filling Cu vacancies through substitutional diffusion—a consequence of the similar atomic sizes of Cu and Zn and the high room-temperature solubility of Zn in Cu (~27 at.%) [13,14].

IMC growth retardation in SAC/Cu-Zn joints arises from two concurrent mechanisms. First, reduced Cu chemical potential at the Cu-Zn surface lowers the thermodynamic driving force for Cu dissolution [15,16]. Second, Zn substitution within Cu_6_(Sn,Zn)_5_ suppresses grain boundary diffusion—the dominant solid-state growth pathway—thereby reducing effective Cu diffusivity [12]. A critical consequence is the complete suppression of Cu_3_Sn and Kirkendall voids. This eliminates the lowest-energy crack path at the wetting layer interface. Under drop impact loading, the high strain rate renders the solder effectively rigid and concentrates stress in the brittle IMC region. Crack propagation is therefore forced into the Cu_6_(Sn,Zn)_5_ interior, rather than along the void-laden Cu_3_Sn/Cu interface that dominates failure in the SAC/Cu system. The characteristically rough Cu_6_(Sn,Zn)_5_/Cu-Zn interface—arising from differential dissolution rates of Cu and Zn during reflow—further impedes interfacial crack advance by increasing the effective fracture energy per unit area [17]. Together, these factors yield a drop impact characteristic life (N_50_) approximately 2.3× higher than for bare Cu specimens [17].

Prior studies have established a thorough mechanistic understanding of SAC/Cu-Zn interfacial behavior and confirmed its reliability superiority over bare Cu. However, a practically important comparison remains unexplored. The surface finish that Cu-Zn must ultimately displace is not bare Cu, but ENIG—the predominant surface finish in advanced packaging and the industrially relevant benchmark for any emerging alternative. In all prior studies, bare Cu served as the sole reference. How SAC/Cu-Zn joints perform relative to SAC/ENIG joints under equivalent aging and impact conditions has not been addressed. This gap is significant. Unlike the Cu-Zn system, ENIG joints contain a P-rich brittle interlayer at the (Cu,Ni)_6_Sn_5_/Ni(P) boundary. This layer grows continuously during aging and provides a secondary fracture path independent of IMC thickness. Whether the absence of this layer in Cu-Zn joints constitutes a sufficient reliability advantage to outweigh the lower diffusion barrier efficiency of Zn relative to Ni has never been examined. No prior study has addressed this question through systematic fractographic comparison.

The present study directly compares the interfacial microstructure evolution and high-speed ball shear reliability of SAC305/Cu-Zn and SAC305/ENIG solder joints, shifting the benchmark from bare Cu to the industrially dominant Ni-based surface finish. Cu-20Zn alloy wetting layers were electroplated from a neutral pyrophosphate-based bath and compared with ENIG using SAC305 solder balls. Specimens were reflowed and subjected to isothermal aging at 180 °C for up to 250 h. Interfacial microstructure evolution was characterized by SEM/EDS cross-sectional analysis. Impact reliability was assessed by high-speed ball shear testing at 1 m/s. Failure mechanisms were analyzed through fractography and fracture mode classification.

Accordingly, the central hypothesis of this study is stated explicitly as follows: the interfacial fracture mechanism—specifically, the presence or absence of a secondary brittle interlayer—rather than the absolute IMC thickness, is the primary determinant of high-speed shear reliability. The objective of this work is to test this hypothesis through a direct, fractography-based comparison of SAC305/Cu-Zn and SAC305/ENIG joints under identical aging and impact conditions.

## 2. Materials and Methods

### 2.1. Test Vehicle and Specimen Preparation

**Figure 1:**
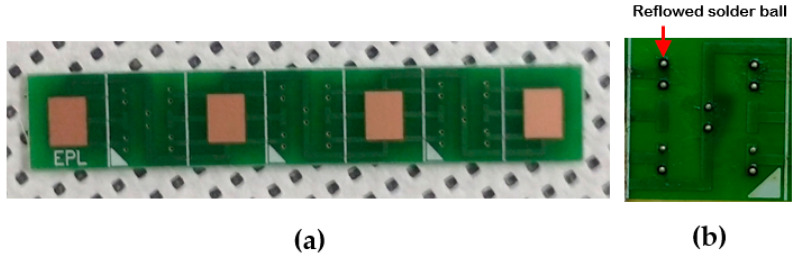
Optical images of the FR-4 substrate test vehicle: (**a**) bare substrate prior to surface finish, showing the solder mask defined (SMD) pad array with a pad opening diameter of 230 µm; (**b**) assembled specimen after SAC305 solder ball reflow.

**Figure 2:**
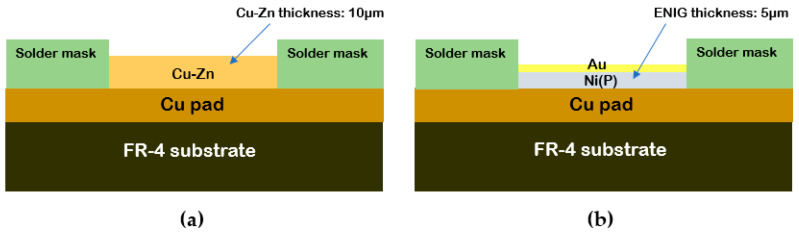
Schematic cross-sections of the two specimen types: (**a**) SAC305/electroplated Cu-20Zn, and (**b**) SAC305/ENIG. Layer thicknesses are indicated for each wetting layer configuration.

A flame resist-4 (FR-4) substrate was used as the test vehicle in this study. The substrate had a photoimageable solder resist (PSR) thickness of approximately 18 μm, a solder mask defined (SMD) pad configuration, and a pad opening diameter of 230 μm (Figure 1). Two types of wetting layers were prepared on the Cu pads for comparative evaluation: electroplated Cu-Zn and ENIG. The cross-sectional schematics of each specimen type is illustrated schematically in Figure 2.

For the electroplated Cu-Zn specimens, Cu-20Zn alloy was deposited onto the Cu pad by electroplating from a pyrophosphate-based bath. The bath composition consisted of CuSO_4_, ZnSO_4_, potassium pyrophosphate (K_4_P_2_O_7_), and HCl at concentrations of 0.02, 0.2, 0.9, and 0.12 mol/L, respectively. Electroplating was performed at a current density of 5 mA/cm^2^ and a bath temperature of 24 °C, resulting in a nominal Cu-Zn surface finish thickness of approximately 10 μm with a standard deviation of 1 μm.

For the ENIG specimens, electroless Ni(P) and immersion Au layers were sequentially deposited on the Cu pad according to a standard ENIG process. The resulting Ni(P) layer thickness was approximately 5 μm with a standard deviation of 0.35 μm, with a phosphorus content of approximately 7 wt%, and the Au layer thickness was approximately 0.1 μm with a standard deviation of 0.01 μm.

After plating, all specimens were rinsed with [deionized water/dilute H_2_SO_4_ solution] and dried to remove residual bath chemistry before solder ball assembly. Surface finish compositions, thicknesses, and deposition conditions for both specimen types are summarized in Table 1.

It should be noted that electroplated Cu-Zn wetting layers are susceptible to surface oxidation. The relatively high chemical activity of Zn can degrade solderability if the surface is left unprotected before solder ball assembly. In practical manufacturing, an organic solderability preservative (OSP) coating is therefore typically applied immediately after electroplating to inhibit oxidation during storage and handling. In the present study, all Cu-Zn specimens were cleaned with dilute H_2_SO_4_ solution immediately before flux application and solder ball assembly to remove surface oxides. Solder ball reflow was performed without OSP treatment to isolate the effect of Cu-Zn wetting layer composition on interfacial microstructure and reliability. This approach is consistent with laboratory-scale protocols adopted in previous Cu-Zn reliability studies. Solder joint quality was confirmed by optical inspection prior to testing.

### 2.2. Solder Ball Assembly and Thermal Aging

SAC305 (Sn-3.0Ag-0.5Cu) solder balls with a diameter of 300 μm (MM series, MK ELECTRON Co., Ltd., Yongin-si, Republic of Korea) were assembled on both types of specimens. Prior to solder ball attachment, all specimens were cleaned with 10% H_2_SO_4_ in deionized (DI) water to remove surface oxides, followed by application of a water-soluble flux on the specimens. The solder balls were then placed onto the flux-coated pads and reflowed using a reflow oven with the following thermal profile: a peak temperature of 260 °C and a time above liquidus (TAL, 217 °C) of (30 s). A minimum of (n = 10) specimens per a wetting layer condition were prepared to ensure statistical reliability.

The assembled specimens were subsequently subjected to isothermal aging in a reflow oven at 180 °C for up to 250 h to examine interfacial microstructure evolution. Specimens were extracted at aging intervals of (0, 50, 100, 250) h for cross-sectional analysis.

### 2.3. Microstructural Characterization and Mechanical Testing

Cross-sectional specimens for SEM observation were prepared by cold mounting in epoxy resin. Mechanical grinding was performed sequentially using SiC abrasive papers of 320, 600, 1200, and 2400 grit, followed by final polishing with a 1 μm aluminum oxide suspension. Soft etching with dilute sulfuric acid was applied to reveal the IMC morphology.

Interfacial microstructure was characterized using a scanning electron microscope (SEM; JEOL JSM-6300, JEOL, Tokyo, Japan) equipped with energy dispersive spectroscopy (EDS; Oxford 6699, Oxford Instruments, Oxford, UK) for elemental analysis. Cross-sectional specimens were examined in both secondary electron (SE) and backscattered electron (BSE) imaging modes to identify IMC phases and fracture surface morphology, respectively. EDS point analysis and mapping were performed to confirm the composition of interfacial IMC phases.

IMC thickness was quantified using image analysis software (Image-Pro Plus 6.0; Media Cybernetics, Rockville, MD, USA). The average IMC thickness was calculated by dividing the cross-sectional area of the IMC layer by its horizontal length, both measured from SEM micrographs. The pixel-to-length conversion factor was calibrated from the scale bar embedded in each SEM image.

**Figure 3:**
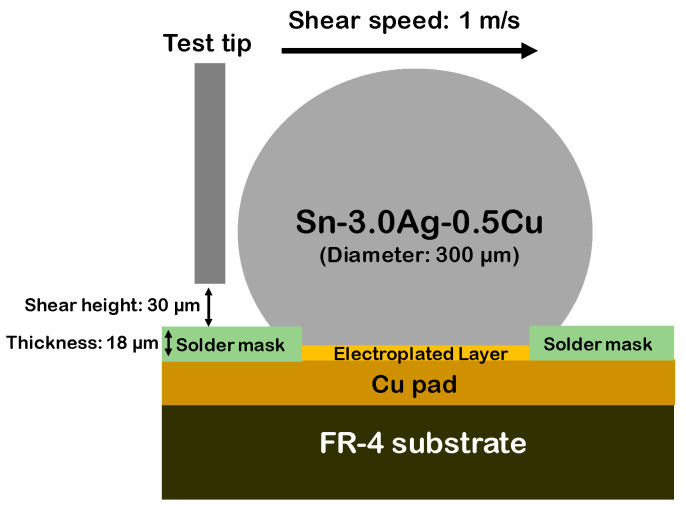
Schematic illustration of the high-speed ball shear test configuration.

Impact reliability was evaluated using a high-speed ball shear tester (DAGE 4000HS: Nordson DAGE, Aylesbury, UK) at a shear speed of 1 m/s and a shear height of 30 μm, as illustrated in Figure 3. A minimum of 10 specimens per condition were tested. The *t*-test for shear force revealed a statistically significant difference in the as-reflowed condition (0 h, *p* = 0.04), whereas no significant difference was observed after aging (50 h, *p* = 0.507; 100 h, *p* = 0.283; 250 h, *p* = 0.455). After shear testing, fracture surfaces were examined by SEM-BSE to identify the failure mode. Fracture modes were classified into three categories based on the brittle fracture area fraction determined from SEM-BSE fractographic analysis: ductile (0–25%), mixed (25–75%), and brittle (75–100%). The brittle fracture area refers to the IMC- and/or P-rich layer-dominated region of the fracture surface, as distinguished from the ductile solder-dominated region.

## 3. Results

### 3.1. Interfacial IMC Phase Identification

**Figure 4:**
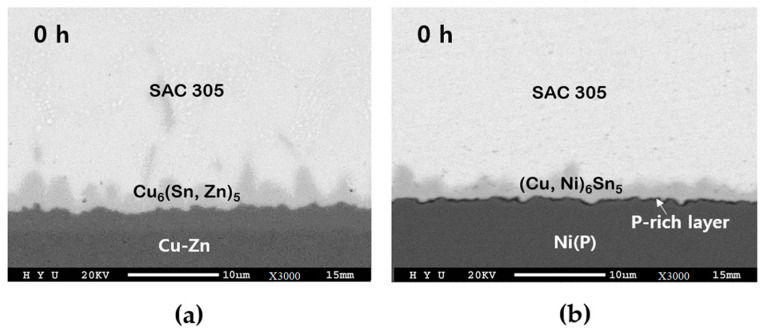
SEM-BSE images showing cross-sections of both specimen types: (**a**) SAC305/Cu-20Zn, and (**b**) SAC305/ENIG.

Figure 4 presents cross-sectional BSE images of the SAC305/Cu-Zn and SAC305/ENIG solder joint interfaces in the as-reflowed condition (0 h). At the SAC305/Cu-Zn interface, a continuous IMC layer was formed immediately after reflow. EDS point analysis confirmed that this phase corresponds to Cu_6_(Sn,Zn)_5_, in which Zn partially substitutes for Sn on the Sn sublattice of η-Cu_6_Sn_5_. The IMC morphology was scallop-like, consistent with liquid-state diffusion-controlled nucleation and growth during reflow. No secondary Cu-Zn intermetallic phases (e.g., CuZn, Cu_5_Zn_8_) were observed at the wetting layer/IMC boundary, confirming that the Zn content of the Cu-20Zn layer was insufficient to form a distinct Zn-rich phase under the given reflow conditions. The initial IMC thickness at the SAC305/Cu-Zn interface was approximately 2.5 μm.

At the SAC305/ENIG interface, EDS analysis identified (Cu,Ni)_6_Sn_5_ as the dominant IMC phase immediately after reflow, as indicated in Figure 4. In the as-reflowed SAC305/ENIG specimen (Figure 4b), a thin, continuous dark-contrast band was observed at the (Cu,Ni)_6_Sn_5_/Ni(P) boundary in BSE imaging. This layer is attributed to a P-rich layer, which forms as a byproduct of electroless Ni deposition: as Ni is selectively consumed into the IMC during reflow, P accumulates at the reaction front and is rejected from the growing (Cu,Ni)_6_Sn_5_ phase, forming a Ni-Sn-P transition layer beneath the IMC. The IMC morphology was also scallop-like, with an initial IMC thickness of approximately 1.94 μm.

### 3.2. IMC Growth Kinetics During Isothermal Aging

**Figure 5:**
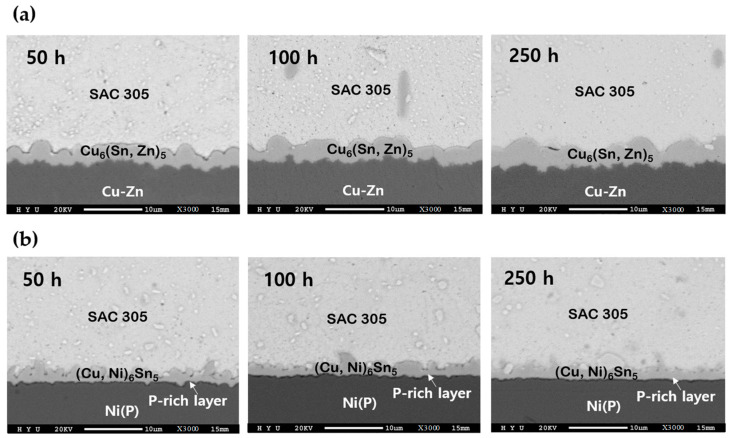
SEM-BSE images showing IMC formation mechanisms during isothermal aging with reflowed SAC305: (**a**) Cu-Zn and (**b**) ENIG wetting layers.

**Figure 6:**
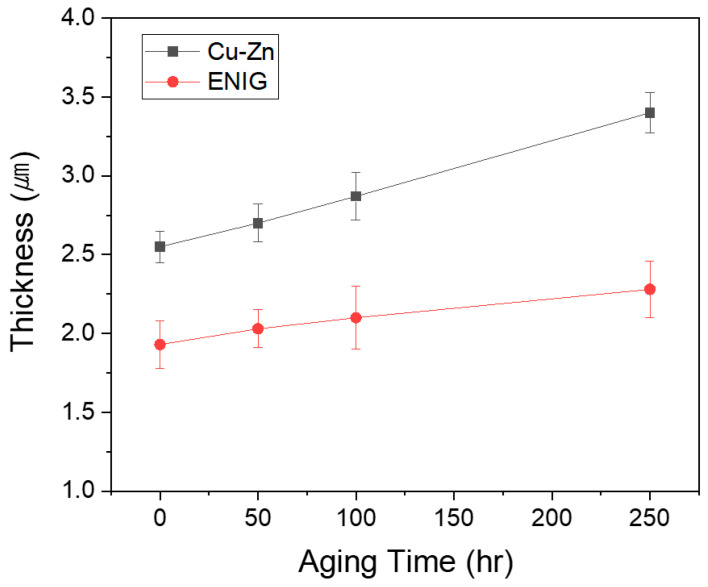
IMC thickness as a function of isothermal aging time at 180 °C for SAC/Cu-Zn and SAC/ENIG solder joints.

Figure 5 presents cross-sectional BSE images showing the interfacial microstructure evolution of SAC305/Cu-Zn and SAC305/ENIG solder joints after isothermal aging at 180 °C for 50, 100, and 250 h. Figure 6 plots the IMC layer thickness as a function of aging time for both specimen groups (SAC305/Cu-Zn, and SAC305/ENIG).

At the SAC305/Cu-Zn interface, a single Cu_6_(Sn,Zn)_5_ IMC layer was identified throughout the entire aging sequence, with no evidence of a distinct Cu-Zn binary intermetallic compound at the wetting layer surface. The Cu_6_(Sn,Zn)_5_ layer thickened progressively with aging time, and the IMC morphology transitioned from an initial scallop-like profile toward a progressively more planar configuration with increasing aging duration. The upper SAC305/Cu_6_(Sn,Zn)_5_ interface became progressively smoother with aging time, consistent with scallop coarsening and lateral merging. In contrast, the lower Cu_6_(Sn,Zn)_5_/Cu-Zn interface retained a rough, irregular morphology throughout aging. This interfacial roughness was established during reflow by the differential dissolution rates of Cu and Zn, and did not evolve significantly with increasing aging time.

At the SAC305/ENIG interface, a (Cu,Ni)_6_Sn_5_ IMC layer formed as the primary reaction product between the SAC305 solder and the electroless Ni(P) layer. A distinct P-rich layer was consistently observed between the (Cu,Ni)_6_Sn_5_ IMC and the underlying ENIG substrate at all aging durations, consistent with the well-established Ni_3_P formation mechanism during Ni consumption. The (Cu,Ni)_6_Sn_5_ IMC layer exhibited a comparatively planar and continuous morphology throughout aging, in contrast to the scallop-to-planar transition observed at the Cu-Zn interface. The P-rich layer remained discernible even after 250 h of aging, indicating that Ni diffusion through the P-rich layer was the rate-limiting step governing interfacial reaction kinetics at the SAC305/ENIG interface.

Figure 6 presents IMC layer thickness as a function of isothermal aging time at 180 °C for SAC305/Cu-Zn and SAC305/ENIG solder joints. Both interfaces showed monotonic IMC growth with increasing aging time. However, the growth rate and absolute thickness differed significantly between the two surface finishes.

At the SAC305/Cu-Zn interface, the Cu_6_(Sn,Zn)_5_ layer thickness increased from approximately 2.50 μm in the as-reflowed condition to 2.70 μm at 50 h, 2.90 μm at 100 h, and 3.41 μm at 250 h. The total growth increment over 250 h was approximately 0.90 μm. The relatively steep slope of the Cu-Zn curve reflects a higher IMC growth rate compared to ENIG. This is attributable to faster Cu diffusivity through Cu_6_(Sn,Zn)_5_ relative to Ni diffusion through (Cu,Ni)_6_Sn_5_.

At the SAC305/ENIG interface, the (Cu,Ni)_6_Sn_5_ layer grew considerably more slowly. Thickness increased from approximately 1.94 μm in the as-reflowed condition to 2.02 μm at 50 h, 2.08 μm at 100 h, and 2.25 μm at 250 h. The total growth increment was only approximately 0.30 μm. This suppressed growth is consistent with the known role of the Ni(P) layer as a diffusion barrier. The Ni(P) layer retards the upward flux of Cu from the underlying Cu pad to the IMC/solder reaction front, thereby limiting (Cu,Ni)_6_Sn_5_ thickening [18,19].

Over the 0–250 h window, the IMC thickness was fitted to the power-law relation, x = x_0_ + k·t^n^. The fitted parameters were k ≈ 0.0057 μm·h^−n^, n ≈ 0.9 (R^2^ ≈ 0.999) for SAC305/Cu-Zn and k ≈ 0.0028 μm·h^−n^, n ≈ 0.85 (R^2^ ≈ 0.999) for SAC305/ENIG. For both interfaces, the exponent lies close to unity, indicating an approximately linear thickness–time dependence over this short window; the limited aging duration does not capture the parabolic (diffusion-controlled) regime expected at longer times. The larger total IMC increment at the Cu-Zn interface (~0.9 vs. ~0.3 μm over 250 h) reflects the absence of a Ni(P) diffusion barrier, and this long-term behavior is identified as future work. Over extended service times, IMC growth is expected to transition to the parabolic (diffusion-controlled) regime and to progressively consume the bulk solder; in the limiting case, the joint would approach a fully intermetallic state, which would substantially reduce its shear strength.

### 3.3. High-Speed Ball Shear Strength

**Figure 7:**
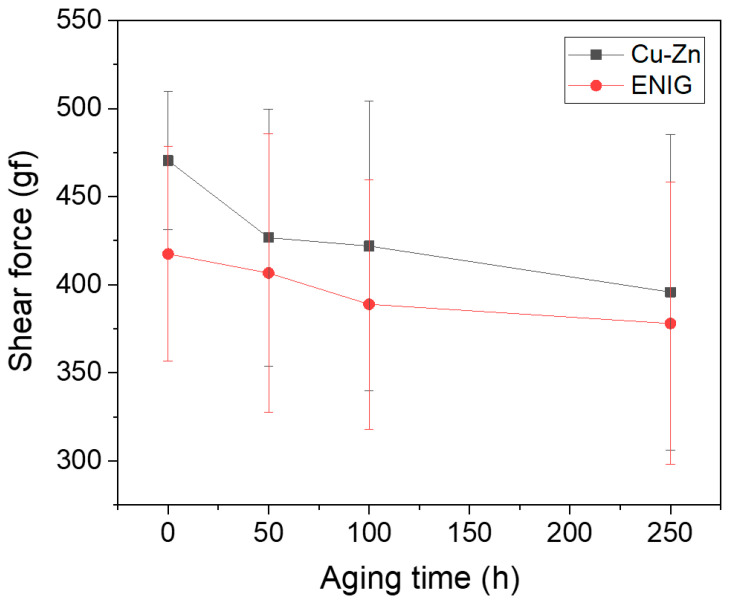
High-speed ball shear strength of SAC305/Cu-Zn and SAC305/ENIG solder joints as a function of isothermal aging time at 180 °C.

Figure 7 presents high-speed ball shear strength of SAC305/Cu-Zn and SAC305/ENIG solder joints as a function of isothermal aging time at 180 °C. In the as-reflowed condition (0 h), the SAC305/Cu-Zn group showed a slightly higher mean shear force (~470 ± 50 gf) than the SAC305/ENIG group (~417 ± 54 gf). Although the difference was significant only in the as-reflowed condition and not significant after aging, the Cu-Zn group consistently maintained higher shear strength across all aging conditions.

With increasing aging time, both groups showed a decreasing trend, but with different characteristics. The SAC305/Cu-Zn group decreased from ~470 gf at 0 h to ~425 gf at 50 h, ~420 gf at 100 h, and ~397 gf at 250 h. The most notable drop occurred between 0 h and 50 h, after which the shear strength remained relatively stable. The total reduction over 250 h was approximately 15%. The SAC305/ENIG group showed a more gradual but continuous decrease, from ~417 gf at 0 h to ~405 gf at 50 h, ~388 gf at 100 h, and ~378 gf at 250 h—a total reduction of approximately 9%.

At 250 h, SAC305/Cu-Zn joints (~397 gf) retained higher shear strength than SAC305/ENIG joints (~378 gf). However, the gap between the two groups narrowed with aging time, as the Cu-Zn group experienced a larger absolute strength reduction. This suggests that the initial shear strength advantage of the Cu-Zn surface finish diminishes under prolonged thermal aging. The large standard deviations observed across all conditions are characteristic of high-speed shear testing, which is sensitive to local variations in solder joint geometry and fracture mode.

### 3.4. Fracture Mode Analysis

**Figure 8:**
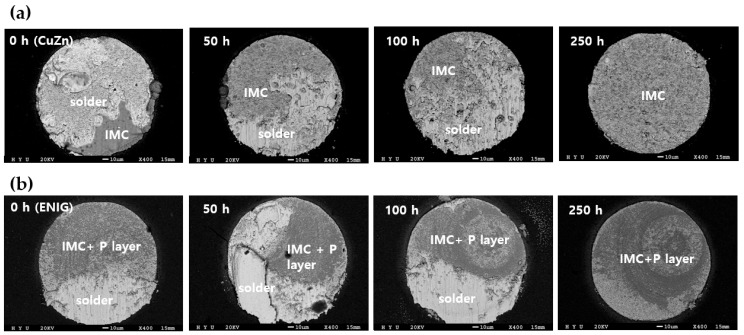
SEM-BSE fracture surface images of (**a**) SAC/Cu-Zn and (**b**) SAC/ENIG solder joints after high-speed shear testing at 0, 50, 100, and 250 h.

**Figure 9:**
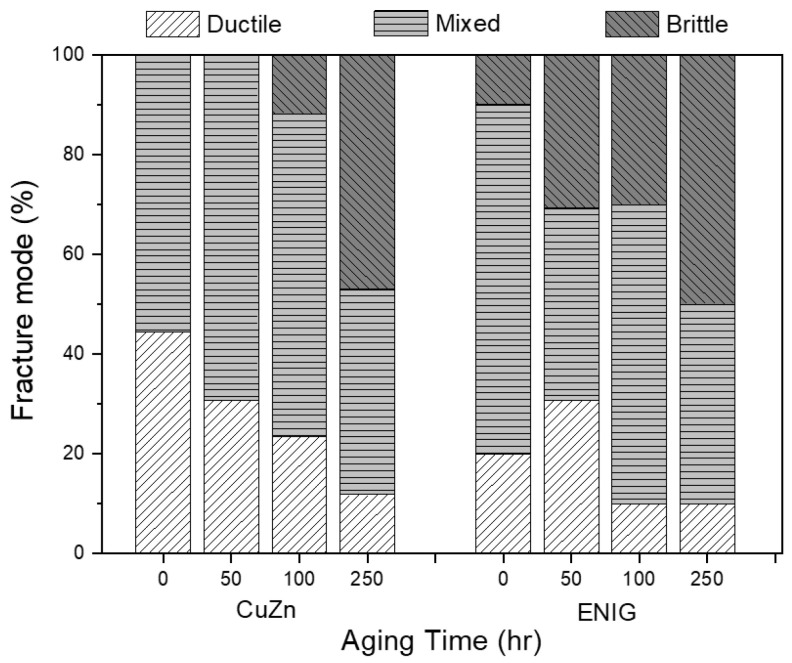
Fracture mode distribution of SAC/Cu-Zn and SAC/ENIG solder joints as a function of aging time at 180 °C, quantified by fractographic area fraction analysis.

Figure 8 presents SEM-BSE fracture surface images of all specimen groups following high-speed shear testing at 0, 50, 100, and 250 h of aging. Figure 9 quantifies the fracture mode distribution—ductile, mixed, and brittle—as a function of aging time for SAC305/Cu-Zn and SAC305/ENIG solder joints.

For SAC305/Cu-Zn joints, fracture in the as-reflowed condition (0 h) occurred through a combination of IMC and solder regions. The fracture mode was entirely ductile–mixed (0% brittle, ~45% ductile, ~55% mixed). At 50 h, a similar mixed fracture mode was maintained with no brittle fracture contribution (0% brittle, ~30% ductile, ~70% mixed). At 100 h, brittle fracture emerged at approximately 12%. This reflects the onset of IMC-dominated crack propagation as the Cu_6_(Sn,Zn)_5_ layer thickened. At 250 h, the brittle fracture ratio increased to approximately 47%, with cracks propagating predominantly within the Cu_6_(Sn,Zn)_5_ IMC interior. Importantly, no secondary brittle interlayer analogous to the P-rich layer in ENIG joints was observed at any aging duration. At 250 h, the brittle fracture ratio of SAC305/Cu-Zn joints (~47%) remained substantially lower than that of SAC305/ENIG joints (~75%) at the same aging duration.

For SAC305/ENIG joints, the fracture surface in the as-reflowed condition (0 h) showed a predominantly mixed morphology comprising IMC- and P-rich layer regions (~10% brittle, ~80% mixed, ~10% ductile). At 50 h, the brittle fracture ratio increased to approximately 35%. This reflects the onset of preferential crack propagation along the P-rich interlayer. The ductile fraction also increased slightly; the present data do not allow a definitive explanation of this small change, which is partly within the scatter of the fractographic classification. At 100 h, fracture became strongly concentrated at the IMC/P-rich layer boundary, with the brittle fracture ratio increasing markedly to approximately 70%. At 250 h, the brittle fracture ratio reached approximately 75%. This is consistent with progressive thickening and lateral continuity of the P-rich interlayer, which increasingly confined crack propagation to this low-toughness region.

**Figure 10:**
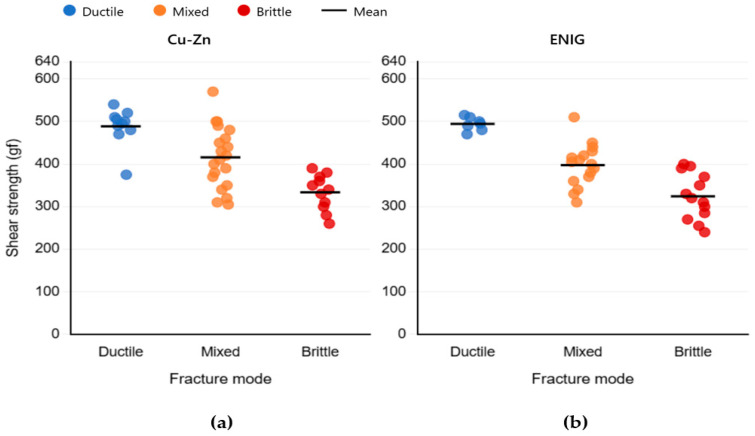
Shear strength distribution as a function of fracture mode for SAC305 solder joints on (**a**) Cu-Zn and (**b**) ENIG surface finishes.

The fracture mode distributions described above directly govern the mechanical performance of each solder joint system. Figure 10 presents shear strength distributions classified by fracture mode for SAC305/Cu-Zn and SAC305/ENIG joints, aggregating all aging conditions. A consistent decrease in shear strength with increasing fracture brittleness was observed across both surface finishes. Because fracture mode is a categorical classification (ductile, mixed, brittle) rather than a continuous variable, this relationship is presented as a grouped trend supported by the per-group mean strengths in Figure 10 rather than as a continuous correlation coefficient. Joints exhibiting ductile fracture yielded the highest shear strengths, followed by mixed-mode joints, while brittle fracture joints showed the lowest values. This trend directly reflects the reduced energy dissipation associated with crack propagation through the stiff IMC or P-rich interlayer, as opposed to the compliant solder matrix.

For SAC305/Cu-Zn joints, ductile fracture specimens showed a mean shear strength of approximately 489 gf. This is consistent with fracture occurring predominantly within the solder bulk. The relatively low scatter (SD = 44.5 gf) reflects the homogeneity of solder bulk fracture prior to extensive IMC growth. Mixed-mode specimens exhibited a broader distribution with a mean of approximately 416 gf. The increased scatter reflects the growing contribution of IMC-interior fracture as the Cu_6_(Sn,Zn)_5_ layer thickened with aging. Brittle fracture specimens showed a mean of approximately 334 gf with moderate scatter. This is indicative of stochastic crack initiation within the IMC layer, consistent with the onset of brittle fracture observed at 100 h and its marked increase at 250 h.

For SAC305/ENIG joints, a comparable trend was observed. However, the P-rich interlayer—which grew progressively in thickness and lateral continuity with aging—drove a monotonic increase in brittle fracture ratio from approximately 35% at 50 h to 75% at 250 h. Brittle fracture specimens of ENIG joints showed a mean shear strength of approximately 324 gf. This is attributable to crack propagation confined to the low-toughness P-rich interlayer with minimal plastic energy absorption.

To quantitatively confirm the fracture-mode/strength relationship shown in Figure 10, the per-mode strength distributions were compared using a non-parametric test (unequal group sizes; normality not assumed). Fracture mode had a statistically significant effect on shear strength for both surface finishes (ENIG: *p* = 1.8 × 10^−5^; Cu-Zn: *p* = 5.4 × 10^−5^). All three modes differed significantly for both surface finishes, in the order ductile > mixed > brittle (ENIG: ductile–mixed *p* = 2.9 × 10^−2^, mixed–brittle *p* = 2.4 × 10^−2^, ductile–brittle *p* = 1.2 × 10^−5^; Cu-Zn: ductile–mixed *p* = 4.6 × 10^−2^, mixed–brittle *p* = 2.4 × 10^−2^, ductile–brittle *p* = 2.9 × 10^−5^). These results quantitatively confirm that increasing fracture brittleness is associated with a statistically significant reduction in shear strength.

Taken together, the data in Figure 10 quantitatively corroborate the fracture mode evolution documented in Figure 9. The earlier and more abrupt brittle transition in SAC305/ENIG joints—particularly at 50 h—translates directly into greater mechanical degradation at intermediate aging stages compared to SAC305/Cu-Zn joints. This divergence persists despite the convergence of brittle fracture fractions at longer aging durations.

## 4. Discussion

### 4.1. Effect of Wetting Layer Composition on IMC Growth Mechanism

The distinct IMC phases formed at the SAC305/Cu-Zn and SAC305/ENIG interfaces—Cu_6_(Sn,Zn)_5_ and (Cu,Ni)_6_Sn_5_, respectively—arise from fundamentally different interfacial reaction mechanisms governed by wetting layer composition.

At the SAC305/Cu-Zn interface, Zn from the wetting layer partitions preferentially onto the Sn sublattice sites of η-Cu_6_Sn_5_, forming Cu_6_(Sn,Zn)_5_ without altering the hexagonal symmetry of the parent phase [15,20]. The retarded IMC growth in SAC305/Cu-Zn joints relative to SAC305/bare Cu joints is attributed to two concurrent effects. First, the reduced Cu chemical potential at the Cu-Zn surface lowers the thermodynamic driving force for Cu dissolution into the molten solder during reflow and for solid-state Cu diffusion during aging [15,16]. Second, Zn incorporation within the Cu_6_(Sn,Zn)_5_ lattice introduces localized lattice distortion. This narrows the effective diffusion channels for Cu atoms through the IMC layer, reducing effective Cu diffusivity and retarding further IMC thickening during solid-state aging [21].

The rough Cu_6_(Sn,Zn)_5_/Cu-Zn interface morphology observed after prolonged aging (Figure 5a) is attributed to selective Zn depletion from the wetting layer surface. During solid-state aging, Zn preferentially partitions toward the solder/IMC interface owing to its higher activity coefficient relative to Cu in dilute Cu-Zn solid solutions. This generates local compositional gradients at the wetting layer surface [16]. The resulting non-uniform recession of the Cu-Zn surface produces the rough interfacial morphology observed after prolonged aging. Notably, Cu_3_Sn formation and Kirkendall void nucleation—consistently observed at the SAC305/bare Cu interface—were effectively suppressed at the SAC305/Cu-Zn interface. This further demonstrates the beneficial role of Zn in stabilizing the interfacial microstructure [15]. This roughening behavior is intrinsic to the Cu-Zn system and is not observed in ENIG joints, where the Ni(P) layer presents a compositionally distinct and structurally stable reaction boundary.

Prior studies established the Cu-Zn mechanism relative to bare Cu. The present study extends this comparison to ENIG. At the SAC305/ENIG interface, (Cu,Ni)_6_Sn_5_ forms as the dominant IMC phase because Cu dissolved from the SAC305 solder (nominally 0.5 wt.%) preferentially stabilizes the η-phase over the competing Ni_3_Sn_4_ (δ) phase. This stabilization is consistent with Sn-Cu-Ni ternary phase equilibria, wherein increasing Cu activity at the interface expands the η-phase field at the expense of the Ni_3_Sn_4_ field [22,23].

The progressive accumulation of the P-rich interlayer during aging results from selective Ni consumption by the advancing IMC front. As Ni is depleted from the electroless Ni(P) layer, P is rejected and accumulates at the reaction boundary, forming a continuously thickening P-rich interlayer beneath the (Cu,Ni)_6_Sn_5_ IMC [1,3]. Once sufficiently thick, this P-rich interlayer itself acts as a secondary diffusion barrier, progressively suppressing further IMC growth [1]. The substantially lower diffusion coefficient of SAC305/ENIG compared to SAC305/Cu-Zn quantitatively reflects the well-established role of Ni(P) as a kinetically superior diffusion barrier. This is attributable to the higher melting point of Ni relative to Cu and the correspondingly lower atomic diffusivity of Ni in Sn-based solders [3].

### 4.2. Relationship Between Fracture Mechanism and Impact Reliability

The central finding of this study is an apparent contradiction between IMC thickness and shear strength. SAC305/Cu-Zn joints possessed a consistently thicker IMC layer than SAC305/ENIG joints throughout aging (Figure 6), yet exhibited equal or superior high-speed ball shear strength at all aging durations (Figure 7). This result demonstrates that IMC thickness alone is not a sufficient predictor of impact reliability under high-speed shear loading [3]. The fracture mode distribution data (Figure 9) reveal that the dominant determinant of reliability is the nature of the interfacial fracture path, which differs fundamentally between the two systems.

In the SAC305/Cu-Zn system, crack propagation under high-speed loading was confined to the interior of the Cu_6_(Sn,Zn)_5_ IMC layer at all aging durations. The fracture mode transitioned progressively from ductile–mixed (0% brittle at 0 h and 50 h) to mixed-brittle (~12% at 100 h) and then to predominantly brittle (~47% at 250 h) as the IMC layer thickened. Throughout this transition, the crack remained within the Cu_6_(Sn,Zn)_5_ interior without involvement of any secondary embrittling interlayer. As a result, the fracture energy per unit crack area is governed primarily by the intrinsic toughness of the Cu_6_(Sn,Zn)_5_ phase itself.

In the SAC305/ENIG system, the P-rich interlayer at the (Cu,Ni)_6_Sn_5_/Ni(P) interface provides an additional, lower-energy fracture path. Its embrittling effect is governed by the thickness and lateral continuity of the P-rich layer itself, rather than by the overlying (Cu,Ni)_6_Sn_5_ IMC thickness. The brittle fracture ratio increased from approximately 10% in the as-reflowed condition to 35% at 50 h, 70% at 100 h, and 75% at 250 h—a total increase of 65 percentage points over the aging sequence. The fractographic data in Figure 8 confirm that this progressive embrittlement is concentrated at the (Cu,Ni)_6_Sn_5_/P-rich layer interface [3]. This represents a fundamentally more deleterious failure locus compared to the IMC-interior fracture observed in SAC305/Cu-Zn joints. At 250 h, the brittle fracture ratio of SAC305/ENIG joints exceeded that of SAC305/Cu-Zn joints by approximately 28 percentage points (~75% vs. ~47%), despite the thinner IMC layer in the ENIG system. Although the absolute shear-strength difference was small and, after aging, not statistically significant, the markedly lower brittle-fracture ratio of the Cu-Zn joints (~47% vs. ~75% at 250 h) indicates a more favorable impact-fracture behavior, at least equivalent to that of ENIG.

Although the absolute shear-strength difference was small and, after aging, not statistically significant, the markedly lower brittle-fracture ratio of the Cu-Zn joints (~47% vs. ~75% at 250 h) indicates a more favorable impact-fracture behavior, at least equivalent to that of ENIG. It should be emphasized, however, that this distinction is largely intrinsic to the high strain-rate regime. The embrittling P-rich interlayer in ENIG joints constitutes a preferential crack path mainly under high strain-rate impact loading, when the solder behaves effectively rigid and stress concentrates at the interfacial region [24]; at low shear speeds, both systems fail predominantly by ductile fracture within the compliant bulk solder, so the surface-finish-dependent brittle path is largely suppressed and the two systems are not expected to differ appreciably [1]. The 1 m/s high-speed shear test thus represents the critical (worst-case) condition for resolving the reliability difference between the two surface finishes. Accordingly, the present conclusions are delimited to the high-speed impact regime; extrapolation of the observed ranking to other loading rates is not claimed, and a systematic evaluation of loading-rate sensitivity is identified as future work (Section 4.4).

### 4.3. Role of the P-Rich Layer as a Preferential Crack Propagation Path

The fracture behavior described in Section 4.2 is underpinned by the physicochemical properties of the P-rich interlayer and its aging-dependent microstructural evolution. The (Cu,Ni)_6_Sn_5_ IMC, despite being brittle, possesses a crystalline structure with finite fracture toughness. The P-rich layer, in contrast, exhibits a columnar crystalline structure and is separated from the overlying (Cu,Ni)_6_Sn_5_ IMC by a thin Ni-Sn-P transition layer containing nanoscale voids. This void-containing Ni-Sn-P transition layer constitutes the lowest-toughness constituent at the SAC305/ENIG interface. It provides an inherently weak fracture locus that operates independently of IMC thickness [2]. As aging progresses from 0 h to 250 h, the P-rich layer becomes increasingly continuous and more clearly resolved in BSE imaging (Figure 5b), consistent with the progressive growth of the Ni–Sn–P/P-rich layer reported for aged SAC/ENIG joints. Although its nanoscale thickness was not directly quantified here—which would require cross-sectional TEM—this increasing continuity is consistent with the progressive rise in brittle fracture ratio documented in Figure 9. Under high-speed impact loading at 1 m/s, the dynamic stress intensity at the interfacial region is substantially amplified [25], and the crack front preferentially follows the lowest-energy propagation path [3]. The continuously expanding void-containing Ni-Sn-P layer consistently provides this path.

In contrast, the SAC305/Cu-Zn system remains entirely free of such a secondary brittle interlayer throughout aging. The brittle fracture ratio of SAC305/Cu-Zn joints increased from 0% at 0 h and 50 h to ~12% at 100 h and ~47% at 250 h. This increase primarily reflects a progressive shift of the crack path from the ductile solder bulk into the Cu_6_(Sn,Zn)_5_ IMC interior as the IMC layer thickens. This is a mechanistically distinct and less deleterious embrittlement pathway. The rough Cu_6_(Sn,Zn)_5_/Cu-Zn interface morphology observed in Figure 5a may provide additional crack path tortuosity. However, direct fractographic evidence for a crack deflection contribution was not obtained in this study. This morphological effect is therefore considered secondary to the absence of a P-rich embrittling interlayer.

In summary, these observations establish that the fracture resistance of the weakest interfacial constituent—rather than the thickness of the dominant IMC layer—is the controlling parameter for high-speed shear reliability in SAC305 solder joints on these surface finish systems.

### 4.4. Implications for Industrial Surface Finish Selection

The results of this study carry direct practical implications for surface finish selection in next-generation high-performance packaging, where both thermal aging resistance and drop/impact reliability are critical design criteria.

ENIG has historically been the dominant PCB surface finish owing to its excellent solderability, flat pad topography, and long shelf life. However, the present study demonstrates that the P-rich layer inherent to the ENIG process constitutes a structural liability under dynamic impact loading. This liability intensifies with thermal aging and is independent of IMC thickness. This finding is particularly relevant for AI server hardware and HPC packaging, where package-to-board interconnections are exposed to elevated operating temperatures that accelerate P-rich layer growth over the product lifetime.

The Cu-Zn wetting layer electroplated from a pyrophosphate-based bath addresses this limitation by entirely precluding P-rich brittle interlayer formation. The resulting Cu_6_(Sn,Zn)_5_ IMC provides a structurally uniform and mechanically adequate fracture path. The slightly higher IMC growth rate of Cu-Zn relative to ENIG does not translate into a reliability penalty under high-speed shear loading. This is demonstrated by the comparable or equivalent shear strength and a lower brittle fracture ratio and lower brittle fracture ratio of SAC305/Cu-Zn joints across all aging conditions.

From a manufacturing standpoint, Cu-Zn electroplating from a neutral pyrophosphate bath offers additional process advantages over ENIG. It eliminates the electroless Ni deposition step and the associated risk of “black pad” defects caused by hyper-corrosion of the Ni(P) surface during immersion Au deposition [26]. It also removes the ferromagnetic insertion-loss concern associated with Ni metallization in high-frequency applications [4,7,8]. The elimination of both the electroless Ni step and the immersion Au deposition is expected to offer cost advantages in high-volume manufacturing, though a detailed process cost analysis is beyond the scope of this study.

One limitation of this study is that aging was limited to 250 h at 180 °C. This may not fully capture long-term IMC growth behavior and reliability degradation relevant to extended service lifetimes. Future work should extend the aging range to ≥500 h, include thermal cycling reliability assessment, provide direct TEM-based quantification of the aging-dependent P-rich layer thickness, and evaluate loading-rate sensitivity. This would more fully establish the long-term viability of Cu-Zn as a competitive alternative to ENIG in demanding packaging environments.

## 5. Conclusions

The interfacial microstructure evolution and high-speed ball shear reliability of SAC305/Cu-Zn and SAC305/ENIG solder joints were systematically investigated after isothermal aging at 180 °C for up to 250 h. This study shifts the reliability benchmark from bare Cu to the industrially dominant ENIG surface finish. The following conclusions are drawn:

(1) At the SAC305/Cu-Zn interface, Cu_6_(Sn,Zn)_5_ was identified as the primary IMC phase after reflow by SEM/EDS analysis. At the SAC305/ENIG interface, (Cu,Ni)_6_Sn_5_ formed as the dominant IMC phase. A P-rich layer was observed at the (Cu,Ni)_6_Sn_5_/Ni(P) boundary in BSE cross-sectional imaging. The IMC morphology at both interfaces was scallop-like in the as-reflowed condition.
(2) The IMC thickness of SAC305/Cu-Zn joints increased from approximately 2.50 μm in the as-reflowed condition to 3.41 μm at 250 h. This consistently exceeded the IMC thickness of SAC305/ENIG joints (1.94–2.25 μm) throughout aging. The Cu-Zn interface showed approximately threefold larger total IMC growth than the ENIG interface over 250 h.
(3) Despite the greater IMC thickness in SAC305/Cu-Zn joints, their high-speed ball shear strength was equivalent or superior to that of SAC305/ENIG joints throughout aging. After aging the shear strengths were statistically equivalent (~397 vs. ~378 gf at 250 h, within the experimental scatter); the Cu-Zn advantage appeared mainly as a lower brittle-fracture ratio rather than as a strength difference. This result demonstrates that IMC thickness alone is not a sufficient predictor of impact reliability. Fractographic analysis revealed that the P-rich interlayer in ENIG joints acts as a preferential crack propagation path under impact loading, driving the brittle fracture ratio to approximately 75% at 250 h and degrading shear strength independently of IMC thickness.
(4) Crack propagation in SAC305/Cu-Zn joints was confined entirely to the Cu_6_(Sn,Zn)_5_ IMC interior at all aging durations, without involvement of a secondary brittle interlayer. The brittle fracture ratio of SAC305/Cu-Zn joints reached approximately 47% at 250 h—28 percentage points lower than that of SAC305/ENIG joints at the same duration. This indicates a more favorable fracture resistance of the Cu_6_(Sn,Zn)_5_-dominated interface. No P-rich or analogous brittle interlayer was observed at any aging condition.
(5) Cu-Zn alloy electroplated from a neutral pyrophosphate-based bath constitutes a highly reliable wetting layer for SAC305 solder joints. By entirely precluding the formation of the P-rich brittle interlayer inherent to ENIG and forming a single-phase Cu_6_(Sn,Zn)_5_ IMC, SAC305/Cu-Zn joints achieve impact reliability equivalent or superior to that of the conventional ENIG surface finish. These results establish Cu-Zn as a viable candidate for next-generation high-performance packaging applications where both thermal aging resistance and impact reliability are critical.

## Figures and Tables

**Table 1:** Bath compositions, applied current densities, and resulting layer thicknesses for the electroplated Cu-20Zn and ENIG surface finish specimens.

Specimen	Bath Composition	Current Density (mA/cm^2^)	Thickness (µm)
Electroplated Cu-20Zn	CuSO_4_ (0.02 M) + ZnSO_4_ (0.2 M) + K_4_P_2_O_7_ (0.9 M) + HCl (0.12 M)	5	10 ± 1
ENIG	Electroless Ni(P) + Immersion Au	N/A	Ni: 5 ± 0.35, Au: 0.1 ± 0.01

## Data Availability

The original contributions presented in this study are included in the article. Further inquiries can be directed to the corresponding author.
